# Early initiation of breastfeeding and associated factors among mothers of aged less than 12 months children in rural eastern zone, Tigray, Ethiopia: cross-sectional study

**DOI:** 10.1186/s13104-019-4718-x

**Published:** 2019-10-21

**Authors:** Senait Gebreslasie Gebremeskel, Tesfay Tsegay Gebru, Berhanu Gebresilassie Gebrehiwot, Hadush Negash Meles, Betell Berhane Tafere, Guesh Welu Gebreslassie, Fisseha Tekulu Welay, Meresa Berwo Mengesha, Desta Abraha Weldegeorges

**Affiliations:** 1 Department of Midwifery, Adigrat University, Adigrat, Ethiopia; 2 Department of Nursing, Adigrat University, Adigrat, Ethiopia; 3 Department of Laboratory, Adigrat University, Adigrat, Ethiopia; 4 Department of Midwifery, Aksum University, Aksum, Ethiopia

**Keywords:** Early initiation, Breastfeeding, Infants, Mothers, Rural eastern zone, Tigray

## Abstract

**Objectives:**

The objectives of this study were to assess early initiation of breastfeeding and associated factors among mothers of aged less than 12 months children in the rural eastern zone, Tigray, Ethiopia.

**Results:**

Totally 803 mother–child pairs were participated in this study with a response rate of 99.25%. Out of this, 787 mothers had ever breastfed their children. Four hundred eighty-seven (61.9%) mothers initiated breastfeeding within 1 h after they gave birth. Mothers having an educational status of primary education were about 2 times more likely to initiate breastfeeding within 1 h of birth [AOR: 1.99, 95% CI 1.36–2.92] and those mothers having secondary education and above were 3.23 times more likely to start breastfeeding [AOR = 3.23, 95% CI 1.99–5.26]. Mothers who had mistimed pregnancy were 58% less likely to initiate breastfeeding within 1 h of birth [AOR: 0.42, 95% CI 0.27–0.65]. On the other hand, mothers who had delivered their child vaginally were 4.6 times more likely to start early initiation of breast feeding [AOR: 4.59, 95% CI 1.99–10.56].

## Introduction

Early initiation of breastfeeding (EIBF) is putting the newborns to feed breast milk within 1 h of birth. Early suckling of the breast milk stimulates milk production and facilitates release of oxytocin. United Nations international children’s emergency fund endorses colostrum as newborns’ perfect food and should be introduced within the first hour after birth [1, 2].

Worldwide, approximately three million babies die every year in their first month of life and 22.3% of neonatal deaths could be prevented by EIBF [3]. Inappropriate feeding practices causes at least 35% of under five children deaths and over two-thirds of these deaths occur during infancy period [4]. Therefore, an estimated 11.6% infant prevented by breastfeeding promotion programs [5]. As a result of this, EIBF is mandatory for the child health.

EIBF practice in low and middle-income countries is low [6]. Even with the known health benefits of EIBF, many countries failed to start EIBF for their newborns [7]. Five million deaths of under five children were reported globally in 2015 and 46% occurred during neonatal period [8]. A systematic review conducted in South America, Asia and Africa found; prevalence of EIBF in Ethiopia ranged from 41.6 to 62.6% [9]. Therefore, counseling during pregnancy is important in order to minimize under-five mortalities attributed to failure and delay to initiate breast feeding early.

Ethiopian government implemented Baby-friendly hospital initiative and community integrated management of childhood illnesses program [10]. Different studies conducted in Ethiopia indicated, EIBF is still low and most of the neonatal mortalities were existed due to delayed initiation of breastfeeding [11–14]. Though, factors associated with EIBF were predominately; socio-economic factors, maternal education, prenatal guidance on breastfeeding and postpartum counseling [15–19].

Most of the studies conducted in Ethiopia were using a small sample size, and conducted in urban areas. Another weakness of these studies were ignored to assess the association with duration of labor, type of pregnancy, parity and complication during pregnancy with EIBF. Therefore, this study was aimed to assess early initiation of breastfeeding and associated factors among mothers of age less than 12 months children in the rural eastern zone, Tigray, Ethiopia.

## Main text

### Methods

Community based cross sectional study design was employed among 809 mothers of children aged less than 12 months in rural eastern zone, Tigray from April to May, 2018. Eastern zone share boundaries with Afar in the East, Southern East Tigray in the south, Central Tigray in the west and Eritrea in the north and have seven woreda/district and two town administration Wukro and Adigrat. Mothers who were critically ill and having serious mental problem were excluded from the study.

The sample size was determined using single population proportion formula by taking prevalence of a study done in Ethiopia (39.6%) [12] and assumptions of 95% confidence interval (CI), 5% margin of error, 10% non-response rate and 2 for design effect.

Of the seven rural woredas of rural eastern zone, Seasi Tsaeda Emba and Ganta Afeshum woredas were selected using lottery method. Of them, 4 tabias from each woreda were selected and sample size was allocated to each tabia proportionally. The data collection tool was adapted from different literatures. A structured questionnaire and face to face interview was conducted and pre-test was also done. Based on the pre-test, questions were revised, and edited. Finally, Tigrigna version questionnaire was used for data collection.

#### Definition of terms

*EIBF* is initiation of breast feeding within 1 h of birth [11, 20].

*Prelacteal feeding* defined as a practice of giving fluid or semisolid food other than breast milk to a child during the first 3 days before the mother’s milk give [21].

*Colostrum avoidance* is squeeze out and throwing of the first breast milk, thick and yellowish milk that is produced in the first 3 days after birth without giving the child [22]. Data was coded, entered and cleaned using Statistical Package for Social Sciences (SPSS) version 22.0. Variables with *P* value ≤ 0.2 in bivariate analysis were entered to multivariate logistic regression. The model of fitness was checked by Hosmer and Lemeshow test. Finally Adjusted Odds Ratio (AOR) with 95% CI and P-value < 0.05 were considered as significantly associated.

### Results

#### Socio-demographic characteristics of participants

A total of 803 mothers were participated in this study which give a response rate of 99.25%. Majority of the mothers were orthodox (99%) in their religion. One-fourth of the mothers education was secondary and above. About one-third of the child birth order found in the 4 to 6th birth order (Table 1).

**Table 1:** Sociodemographic characteristics of mothers in rural eastern zone, Tigray, Ethiopia, 2018 (n = 803)

Variable	Category	Frequency (n)	Percentage (%)
Age of mothers (in years)	15–19	14	1.7
	20–24	208	25.9
	25–29	180	22.4
	30–34	177	22
	≥ 35	224	27.9
Religion	Orthodox	798	99
	Others	8	0.9
Ethnicity	Tigray	791	98.5
	Amhara	12	1.5
Marital status	Single	66	8.2
	Married	727	90.5
	Others	10	1.2
Educational status of mothers	No formal education	318	39.6
	Primary education	285	35.5
	Secondary education and above	200	24.9
Mothers occupation	Housewife	735	91.5
	Daily laborer	20	2.5
	Farmer	26	3.2
	Others	22	2.8
Fathers education	No formal education	231	28.8
	Primary education	333	45.2
	Secondary education and above	172	23.4
Occupation of fathers	Farmer	490	61
	Daily laborer	150	18.7
	Merchant	47	5.9
	Private organization	26	3.2
	Others	34	11.2
Child age (in months)	< 1 month	57	7.1
	1–6 month	476	59.3
	> 6 month	270	33.6
Sex of child	Male	455	56.7
	Female	348	43.3
Family size	≤ 3	417	51.9
	≥ 4	386	48.1
Child birth order	1	189	23.5
	2–3	234	29.1
	4–6	289	36
	> 6	91	11.3
Child birth interval	No previous child	189	23.5
	< 24 months	89	11.1
	≥ 24 months	525	65.4

#### Feeding practices and health service utilization of study participants

Six hundred forty-eight (80.7%) of the respondents had intended pregnancy. Of 796 mothers who had antenatal care (ANC) visit, 21.7% had 2–3 times. Total 787 (98%) mothers were ever breastfed their index child, and 487 (61.9%) of participants were initiated breastfeeding within 1 h for their child (Table 2).

**Table 2:** Feeding practices and health service utilization in rural eastern zone, Tigray, Ethiopia, 2018 (n = 803)

Variable	Alternatives	Frequency (n)	Percentage (%)
Parity	Primi	199	24.8
	Multipara	604	75.2
Gestational age	Preterm	12	1.5
	Term	777	96.8
	Post term	14	1.7
Pregnancy	Intended	648	80.7
	Unintended	39	4.9
	Mistimed	116	14.4
Complication during pregnancy	Presence	50	6.2
	Absence	753	93.8
Place of delivery	Home	42	5.2
	Health institution	761	94.8
Mode of delivery	Cesarean section	28	3.5
	Vaginal delivery	775	96.5
Duration of labor	< 12 h	673	83.8
	≥ 12 h	130	16.2
ANC follow up	Yes	796	99.1
	No	7	0.9
Number of ANC visit (n = 796)	1	6	0.8
	2–3	173	21.7
	≥ 4	617	77.5
Healthcare providers counseling during ANC (n = 796)	Yes	409	51.4
	No	387	48.6
PNC follow up	Yes	100	12.5
	No	703	87.5
Healthcare providers counseling during PNC (n = 100)	Yes	83	83
	No	17	17
Decision maker	Mother	775	96.5
	Healthcare providers	22	2.8
	Others	6	0.7
Ever breastfeed	Yes	787	98
	No	16	2
EIBF (n = 787)	Yes	487	61.9
	No	300	38.1
Colostrum feeding (n = 787)	Yes	669	85
	No	118	15
Pre-lacteal feeding	Yes	198	24.7
	No	605	75.3

Three hundred mothers were delayed to initiate breastfeeding. The most common reason mentioned for delayed initiation of breastfeeding by the mothers was ‘my child was not with me’ in 192 (64) (Additional file 1: Figure S1).

#### Factors associated with early initiation of breastfeeding

Variables with P-value ≤ 0.2 in bivariate analysis were exported into the multivariable logistic regression model. In multi variable logistic regression; mothers’ having primary and secondary education, having mistimed pregnancy and giving birth by vaginal delivery were statistically associated with early initiation of breastfeeding at P-value < 0.05.

Mothers having an educational status of primary education were about 2 times more likely to initiate breastfeeding within 1 h of birth [AOR: 1.99, 95% CI 1.36–2.92] and those mothers having secondary education and above were 3.23 times more likely to start breastfeeding those mothers who had no formal education [AOR = 3.23, 95% CI 1.99–5.26].

Compared to mothers who had intended pregnancy, those mothers who had mistimed pregnancy were 58% less likely to initiate breastfeeding within 1 h of birth [AOR: 0.42, 95% CI 0.27–0.65]. On the other hand, mothers who had delivered their child vaginally were 4.6 times more likely to start EIBF [AOR: 4.59, 95% CI 1.99–10.56] (Table 3).

**Table 3:** Factors associated with early initiation of breastfeeding in rural eastern Tigray, Ethiopia, 2018

Variables	Category	Early initiation of breast feeding		COR (95% CI)	AOR (95% CI)	P-value
		Yes	No			
Educational status of mothers	No formal education	162	151	1	1	
	Primary education	184	93	1.84 (1.32–2.67)	1.99 (1.36–2.92)*	*0.000*
	Secondary education and above	141	56	2.35 (1.60–3.43)	3.23 (1.99–5.26)*	*0.000*
Child birth order	1	118	67	1.80 (1.08–3.00)	0.87 (0.47**–**1.61)	0.66
	2–3	142	86	1.69 (1.03–2.77)	0.95 (0.55**–**1.67)	0.87
	4–6	183	102	1.84 (1.13–2.97)	1.45 (0.87**–**2.41)	0.15
	> 6	44	45	1	1	
Pregnancy	Intended	413	224	1	1	
	Unintended	11	26	2.49 (1.66–3.75)	1.52 (0.71**–**3.22)	0.278
	Mistimed	65	48	3.20 (1.44–7.11)	0.42 (0.27–0.65)*	*0.000*
Mode of delivery	C/S	10	18	1	1	
	Vaginal delivery	477	282	3.05 (1.39–6.69)	4.59 (1.99–10.56)*	*0.000*
Counseling during ANC	Yes	259	141	1.25 (0.94–1.67)	1.15 (0.84**–**1.58)	0.385
	No	154	226	1	1	

* Statistical significance (P < 0.05), 1 = reference, *COR* crude odds ratio

## Discussion

Breastfeeding is an essential primary health care practice for optimal care of a newborn [23]. Therefore, this study aimed to assess early initiation of breastfeeding and associated factors among mothers of age less than 12 months children in rural eastern zone, Tigray, Ethiopia.

This study indicated that, 61.9% of the participants initiated breastfeeding within one hour of child birth. This is consistent with a study conducted in Debre berhan and slightly lower than the findings from Uganda (68.6%) [24], Arsi (67.3%) [25], Motta (78.8%) [26] and Southern Ethiopia (83.7%) [27] but this result is higher than studies from India (38.6%) [28], Tanzania (51%) [29] and North eastern Ethiopia (39.6%) [12]. These dissimilarities could be due to difference in; sociodemographic characteristics, health service utilization, feeding styles, study area and sociocultural practices.

Mothers’ education, type of pregnancy and mode of delivery were statistically associated with EIBF. In which, mothers having secondary education and above were 3.28 times more likely to start breastfeeding than those mothers having no formal education. Similar studies were found from India, South Asia, Gurage, Amibara, and Arsi zone [11, 12, 16, 18, 28]. This justified as, mothers attending formal education might acquire necessary information on proper breastfeeding practices from school setup, read and understand easily concerning breastfeeding promotion materials.

Mothers who had mistimed pregnancy were 58 times less likely to initiate breastfeeding early in comparison with those mothers who had intended pregnancy. No study finding which inline or opposed to this result was found. Women who experienced mistimed pregnancy might miss familial or partner support for good health care seeking behaviour of their children. This result, depression and psychological instability and this leads on higher risk of adverse health outcomes of the newborn and mother.

Mothers who had delivered their child vaginally were 4.6 times more likely to start breastfeeding than those who delivered by cesarean section. This study in line with different studies from South Asia, India, Tanzania, Uganda, Amibara, Debre berhan, Gurage and Ethiopia [11–13, 16, 18, 19, 24, 28, 29]. This might be explained because mothers who deliver their child vaginally are close with their children due to different tasks for the children like immediate new-borne care and skin to skin contact.

## Conclusion

Based on the findings of this study, about two-third of the mothers timely initiated to breast fed their child. Mothers’ primary and secondary education, Mistimed pregnancy and mothers who had delivered their child vaginally were statistically associated with EIBF.

## Limitation

There is a potential recall bias among respondents and the nature of study design could not show seasonal variation and temporal relationship of cause and effect.

## Supplementary information

**Additional file 1: Figure S1.** Reasons for late initiation of breastfeeding among mothers of aged less than 12 months children in rural eastern zone, Tigray, Ethiopia, 2018.

## Data Availability

The datasets during and/or analyzed during the current study available from the corresponding author on reasonable request.

## Abbreviations

ANC: ante natal care
AOR: adjusted odds ratio
CI: confidence interval
EIBF: early initiation of breastfeeding
PNC: post natal care
SPSS: Statistical Package for Social Science
