# Supplementary material for: Early initiation of breastfeeding and associated factors among mothers of aged less than 12 months children in rural eastern zone, Tigray, Ethiopia: cross-sectional study
Source: BMC Res Notes. 2019 Oct 21;12:671. doi: 10.1186/s13104-019-4718-x (PMC6805677; doi:10.1186/s13104-019-4718-x)
Supplement: Supplementary file 1 — Additional file 1: Figure S1. Reasons for late initiation of breastfeeding among mothers of aged less than 12 months children in rural eastern zone, Tigray, Ethiopia, 2018. [file 13104_2019_4718_MOESM1_ESM.docx]

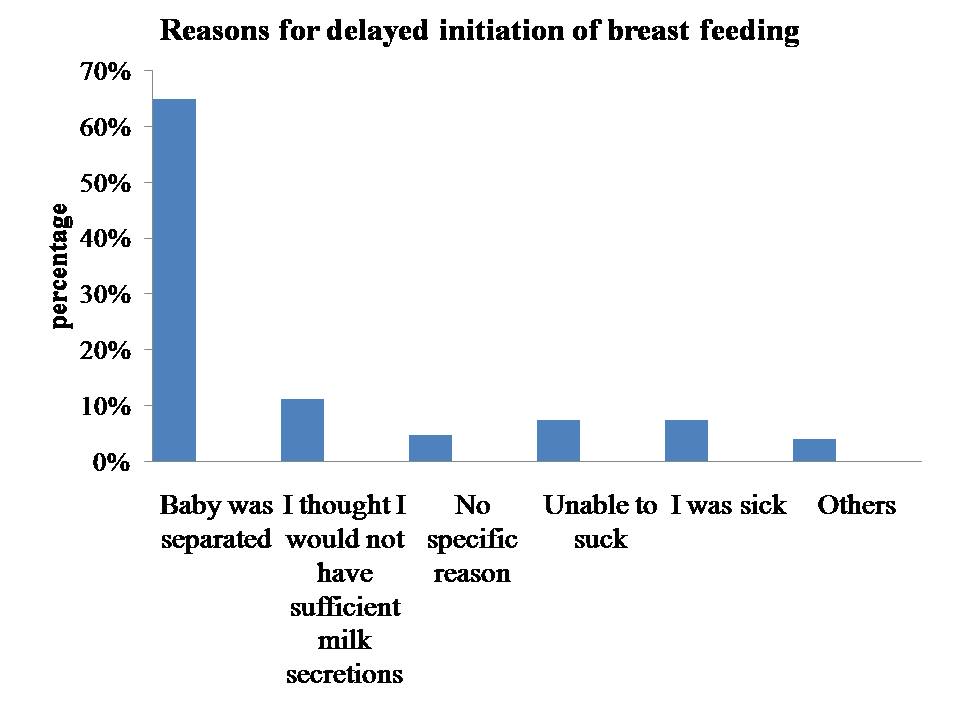


**Additional file 1: Reasons for late initiation of breastfeeding among mothers of aged less than 12 months children in rural eastern zone, Tigrai, Ethiopia, 2018**
